# Feasibility of a Randomized Controlled Trial of Large AI-Based Linguistic Models for Clinical Reasoning Training of Physical Therapy Students: Pilot Randomized Parallel-Group Study

**DOI:** 10.2196/66126

**Published:** 2025-07-23

**Authors:** Raúl Ferrer-Peña, Silvia Di-Bonaventura, Alberto Pérez-González, Alfredo Lerín-Calvo

**Affiliations:** 1 Grupo de Investigación Clínico-Docente sobre Ciencias de la Rehabilitación (INDOCLIN), Centro Superior de Estudios Universitarios La Salle, Universidad Autónoma de Madrid, La Salle 10, Aravaca, Madrid, 28023, Spain, 34 917401980; 2 Departamento de Fisioterapia, Centro Superior de Estudios Universitarios La Salle, Aravaca, Madrid, Spain; 3 Department of Physical Therapy, Occupational Therapy, Rehabilitation and Physical Medicine, Rey Juan Carlos University, Alcorcón, Spain; 4 Grupo de Investigación de Neurociencias Aplicadas a la Neurorehabilitación (GINARE), Neuron, Madrid, Spain

**Keywords:** clinical reasoning, physical therapy education, artificial intelligence, pilot study, feasibility, physical therapy, randomized controlled trial, artificial intelligence-based, linguistic model, physical therapy student, critical skill, large language models, LLM, barriers, facilitator, effectiveness, implementation, physiotherapy

## Abstract

**Background:**

Clinical reasoning is a critical skill for physical therapists, involving the collection and interpretation of patient information to form accurate diagnoses. Traditional training often lacks the diversity of clinical cases necessary for students to develop these skills comprehensively. Large language models (LLMs) like GPT-4 have the potential to simulate a wide range of clinical scenarios, offering a novel approach to enhance clinical reasoning in physical therapy education.

**Objective:**

The aim of the study is to explore the main barriers and facilitators that could be encountered in conducting a randomized clinical trial to study the effectiveness of the implementation of LLM models as tools to work on the clinical reasoning of physical therapy students.

**Methods:**

This pilot randomized parallel-group study involved 46 third-year physical therapy students at La Salle Centre for Higher University Studies. Participants were randomly assigned to either the experimental group, which received LLM training, or the control group, which followed the usual curriculum. The intervention lasted for 4 weeks, during which the experimental group used LLM to solve weekly clinical cases. Digital competencies, satisfaction, and costs were evaluated to explore the feasibility of this intervention.

**Results:**

The recruitment and participation rates were high, but active engagement with the LLM was low, with only 5.75% (5/23) of the experimental group actively using the model. No significant difference in overall satisfaction was found between the groups, and the cost analysis reflected an initial cost of US $1738 for completing the study.

**Conclusions:**

While LLMs have the potential to enhance specific digital competencies in physical therapy students, their practical integration into the curriculum faces challenges. Future studies should focus on improving student engagement with LLMs and extending the training period to determine the feasibility of integrating this tool into physical therapy education and maximize benefits.

## Introduction

Clinical reasoning, a vital skill in physical therapist training, is essential to ensure safe and efficacious practice [1]. This multifaceted process relies on the collection and interpretation of patient information to formulate an accurate diagnostic hypothesis [2]. A crucial part of this process is instant patient feedback, which can help refine hypotheses and tailor interventions according to the patient’s individual needs [3].

However, a significant limitation in the training of physical therapy students is the lack of access to a sufficient variety of clinical cases during their internships. Students often encounter a limited range of clinical conditions during their training, which can limit their ability to develop clinical reasoning skills in a wide range of settings [4]. This lack of diversity in clinical experience can be especially problematic given that physical therapists often encounter a wide range of conditions in their subsequent practice [5]. In Spain, the physical therapy degree follows a competency-based model in which students are expected to progressively develop clinical reasoning skills throughout their education. However, these skills are not always formally assessed beyond traditional written exams, which may not adequately reflect real-world decision-making.

To address these challenges, different pedagogical approaches have been implemented in physical therapy education to enhance clinical reasoning skills. Some of the most widely used methodologies include problem-based learning, case-based discussions, and simulated patient encounters [6]. While these strategies have shown positive results in improving students’ decision-making skills, their implementation often requires significant resources, such as trained facilitators or access to standardized patients, making them challenging to scale [7]. This highlights the need for alternative approaches that can provide physical therapy students with diverse clinical scenarios to enhance their clinical reasoning in a more accessible and structured manner.

One such alternative is the use of artificial intelligence (AI)–based large language models (LLMs), such as GPT-4, which have emerged as promising tools for improving this critical skill in physical therapy students [8].

LLMs can simulate realistic and online clinical cases, allowing students to engage in patient scenarios without the logistical constraints of traditional training methods. Previous studies have shown that LLMs can improve diagnostic accuracy, clinical decision-making, and knowledge retention, particularly in medical and nursing students [9,10].

Despite this potential, the use of LLMs in physical therapy education remains underexplored, and it is unclear whether they can provide similar benefits for clinical reasoning in this field. Moreover, to our knowledge, no studies have directly replicated an existing protocol for integrating LLMs into physical therapy education, making it difficult to assess their impact systematically.

In physical therapy education, the assessment of clinical reasoning is conducted through a combination of formative and summative evaluations. Formative evaluation assesses students’ skills and attitudes throughout their coursework, while summative evaluation consists of final written and oral-practical tests to measure the acquisition of clinical competencies [11].

Although these methods are effective in measuring theoretical knowledge, they may not fully capture the complexity of real-world clinical reasoning. LLMs could help bridge this gap by providing adaptive, interactive case simulations that allow students to refine their decision-making in a controlled, risk-free environment.

While artificial intelligence has already demonstrated benefits in health care applications such as pathology, where it has improved productivity, diagnostic accuracy, cost-efficiency, and staff satisfaction [12], its application in physical therapy remains largely theoretical. More research is needed to determine whether these models can effectively enhance physical therapy students’ clinical reasoning skills in a structured educational setting.

Therefore, the aim of the study is to explore the main barriers and facilitators that could be encountered in conducting a randomized clinical trial to study the effectiveness of the implementation of LLM models as tools to work on the clinical reasoning of physical therapy students.

## Methods

### Ethical Considerations

This study was approved by the local ethics committee of the La Salle University Center for Advanced Studies, Madrid, Spain (CSEULS-PI-002/2025). This trial was registered in clinicaltrials.org (NCT06809634). This study followed the CONSORT guideline for randomized controlled trials (RCTs) [13]. All information and consent forms were provided in an easy-read format, and written informed consent was obtained from all participants. Participants were assured that their personal data would be kept confidential and only used for the purposes of this study. All data were anonymized and stored in accordance with privacy regulations. Participants were informed of their right to withdraw from the study at any time without any consequences. No compensation was provided for participation, but participants were informed of any potential benefits associated with the study outcomes.

### Design

A pilot randomized parallel-group study was conducted in La Salle Centre for Higher University Studies (LCHUS). In total, 46 students from different third-year physical therapy classes participated in the study. These 2 classes were randomly assigned to the experimental or control group using a simple randomization after signing the informed consent by an external researcher who did not participate in the study. Participants in the experimental group received the LLM training in addition to attending course lessons, while the participants in the control group received only the course lessons. The study was carried out between September 2023 and January 2024.

In the context in which this study was carried out (Spain), physical therapists have the capacity to carry out evaluations, formulate functional diagnoses and establish treatment plans without the need for a medical referral in most cases, except in those pathologies that require previous medical intervention, as established in Order CIN 2135/2008 [14]. In this study, students were trained to apply this professional framework in a simulated environment, where they had to [1] formulate diagnostic hypotheses based on the anamnesis and clinical examination of the online patient [2], make a physiotherapeutic diagnosis in line with the WHO’s (World Health Organization) International Classification of Functioning, Disability and Health (ICF) and [3] design a treatment plan according to the patient’s functional assessment of the patient. This approach sought to replicate the physical therapist’s clinical reasoning process in a controlled context, favoring the development of critical skills in clinical decision-making. The trial follows the CONSORT (Consolidated Standards of Reporting Trials) guidelines (the CONSORT checklist is provided in Checklist 1).

### Development of the Theoretical Framework of Learning

The physical therapist’s program at the LCHUS (Autonomous University of Madrid) follows a clinical reasoning approach based on hypothetical-deductive theory and decision-making in real and simulated clinical environments [2,3]. This model integrates 2 main approaches: 1, the hypothetical-deductive model, in which students generate diagnostic hypotheses based on the medical history and clinical examination, contrasting them with new tests to adjust their reasoning [1]; 2, narrative reasoning, which emphasizes the patient’s history and their biopsychosocial context for individual-centered decision-making [15]; and 3, reflective reasoning and metacognition, which encourages self-assessment of clinical thinking through tutorials and structured feedback [10]. The acquisition and consolidation of clinical reasoning in the program is evaluated through a system of formative and summative assessment. Formative assessment is based on observation and feedback during face-to-face and simulated practical, written reflection in learning portfolios and classroom discussion of clinical cases. On the other hand, summative assessment includes structured practical exams, where students must justify their diagnostic and therapeutic reasoning in standardized clinical scenarios, as well as written evaluations of clinical cases and self-assessments using tools such as the Lasater Clinical Judgment Rubric [10].

### Sample Size

This was a feasibility study, so a formal sample size calculation was not required. In total, 46 participants were recruited for the study and allocated in equal numbers in the control and experimental groups.

### Blinding

Students were not blinded to the group allocation because they knew if they had to work with or without using the LLM. Therefore, the person applying the treatment (in this case the lessons) was aware of the group to which each of the participants had been assigned. Assessors were blinded to the assignment of the participants.

### Interventions

After being randomized, for those students belonging to the experimental group (LLM Group), a personal LLM ChatGPT account in version 3.5 was generated for them for a period of 1 month. Using this account, the participants solved a total of 4 clinical cases for 4 weeks, one per week, in which the LLM will serve as a virtual patient, answering the questions that the student asked and based on a physical therapy diagnosis, participants proposed a treatment for the virtual patient. It is important to note that students were able to perform the same clinical case as many times as they wanted, which would be supervised for the researchers after ending the study. No formal training was provided to students on how to use the LLM before the intervention. This decision was intentional, as the aim was to observe how students engaged with the tool naturally, identifying potential barriers and facilitators without prior guidance influencing their interaction.

The control group, on the other hand, carried out the usual practices prescribed by the LCHUS, in which, during the same 4 weeks, they participated in the physical therapy sessions carried out by their practice tutor, intervening with him in the clinical reasoning of the patients treated. In turn, they carried out the same cases as in the experimental group, in written format, which they had to resolve in the same way with the practice tutor.

At the end of the 4 weeks, the students in the experimental and control groups were evaluated with a final clinical case study in written format.

**Figure 1.**
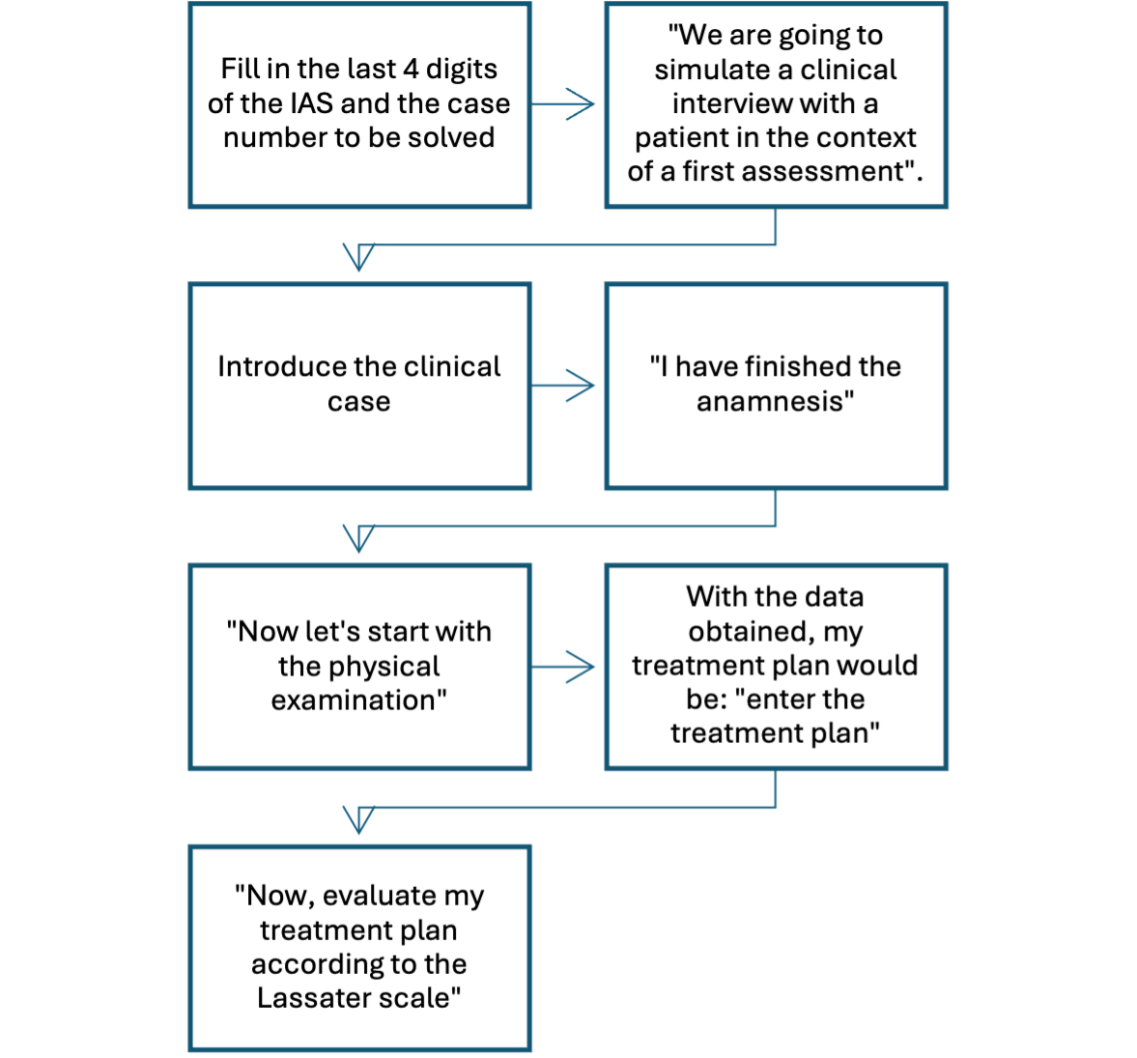
Predefined prompts for clinical cases. Figure developed by the authors based on previous literature on artificial intelligence in physical therapy and medical education [8,16]. IAS: Institutional Academic System.

For the participants in the experimental group to execute the clinical cases, they had to follow a procedure and write predefined prompts in the LLM, which were provided to them via a PDF file. The procedure for the execution of the clinical cases and the prompts entered at the beginning of the cases are shown in Figure 1. Students who did not complete the required four weeks of LLM clinical scenario cases were not included in the final analysis, as their incomplete participation could bias the results. However, their academic performance in the course was assessed through the standard evaluation methods established by the institution, ensuring that all students met the competency requirements of the physical therapy curriculum.

The clinical cases presented in the LLM consisted of a person with low back pain with facet characteristics, a patient with knee pain with characteristics related to chondromalacia patella, a patient with rheumatoid arthritis, and a person presenting to the clinic for postsurgical rehabilitation of a hip prosthesis. An example of the prompts used in the cases and the expected interactions can be found in Multimedia Appendix 1.

### Outcomes

#### Recruitment and Participation

Study recruitment and retention data include the number of students who expressed interest to take part in the study, participants' enrollment, and participants completing treatment.

#### Cost Analysis

The cost analysis of the intervention was calculated based on the cost of the ChatGPT access plan from OpenAI, which provides ChatGPT version 4.0 rather than the free ChatGPT 3.5 account. To accurately capture the effective cost, we also considered the teaching period during which students had access to the program, in alignment with the course calendar.

However, in order to reflect the practical realities when evaluating the feasibility of integrating LLM into teaching practice, it is necessary to consider other associated costs, both direct and indirect. Among the most significant is the need to train instructors who will incorporate this model as part of their teaching methodology. This entails expenses related to instructional hours (eg, introduction to LLM, design and engineering of prompts, and methods for evaluating the information generated), as well as preparation time, rehearsal of the proposed routine or procedure, and the acquisition, comparison, or assessment of the results obtained. Since traditional assessment systems will be modified, it is also important to review interactions with the AI, which may increase workload hours unless automated systems are established to record the data generated.

Likewise, the costs of integrating these models into the academic setting should be assessed, including potential changes to academic guidelines, the provision of necessary resources for students, the required technical support, and relevant training to ensure effective use of the technology. However, the costs associated with integrating these models into teaching practice could reduce future expenses after the initial investment, since in the short term—and once the model is integrated—direct or indirect benefits are expected to be obtained in the long run.

#### Suitability of Outcome Measures

The outcome measurements tool used in the feasibility study was an ad hoc questionnaire for digital competencies [17]. A visual analog scale was used to evaluate the satisfaction of participants with the educative intervention.

The choice to include a digital competencies questionnaire was closely tied to our main objective: evaluating how effectively students could adopt and benefit from an LLM-based intervention. Because the experimental group interacted with ChatGPT, it was crucial to determine whether participants possessed (or could develop) the necessary digital skills to navigate and critically assess AI-generated content.

The questionnaire developed by Montero-Delgado et al (2020) [17] assesses the 26 digital competencies for health care professionals, divided into six domains. Other similar ad hoc questionnaires have shown that it is a valid and reliable tool. For example, in the study carried out in patients with type 1 diabetes by Barajas-Galindo et al [18], the questionnaire was shown to be valid, with a Cronbach α coefficient of .77. By measuring these competencies, we aimed to identify whether insufficient digital literacy might hinder the effective use of AI-based tools and to observe any potential improvements in key digital skills that could support clinical reasoning in physical therapy. The Spanish version of this questionnaire is included in Multimedia Appendix 2.

The visual analog satisfaction scale consisted of a 100 mm line on which the participant had to indicate the final satisfaction with the educational program in a range between very dissatisfied (0 mm) and very satisfied (100 mm).

### Statistical Analysis

Once the data collection period was over, data analysis was carried out using SPSS statistical software version 29.0 (SPSS Inc). A descriptive analysis of the demographic characteristics and the main and secondary variables in the sample was performed, presenting continuous variables as mean SD, while categorical variables are presented as number (n) and percentage (relative frequency, %). Descriptive statistics on recruitment and retention rates, baseline participants' characteristics, and acceptability of the treatment were reported, and differences between groups were examined with *t* and chi-square tests.

## Results

### Study Participants

After randomization, 23 participants were assigned to the LLM group, and 23 participants were included in the control group. Out of 23 participants, there were 17 (73.9%) females in the LLM group and 9 (39.1%) females in the control group. Baseline sociodemographic data for both groups are reported in Table 1. Statistically significant differences were found between groups for gender, nationality, and language variables.

**Table 1.** Baseline characteristics of all randomized participants.

Category		Control	Intervention	*P* value	Rosenthal R
Age, mean (SD)		20.48 (1.78)	21.29 (1.3)	.08^a^	0.257
Nationality, n (%)				.005^b^	0.419
	Spanish	11 (47.8)	20 (87.0)		
	French	12 (52.2)	3 (13.0)		
Language, n (%)				.005^b^	0.419
	Spanish	11 (47.8)	20 (87.0)		
	French	12 (52.2)	3 (13.0)		
Gender, n (%)				.02^b^	0.353
	Man	6 (26.1)	14 (60.9)		
	Woman	17 (73.9)	9 (39.1)		
Educational level, n (%)				—^c^	—
	Bachelor	23 (100)	23 (100)		
Ethnic group, n (%)				.54^d^	0.153
	White	11 (47.8)	11 (47.8)		
	Latin	12 (52.2)	10 (43.5)		
	Asian	0 (0)	1 (4.3)		
	Black	0 (0)	1 (4.3)		
Civil status, n (%)				—	—
	Unmarried	23 (100)	23 (100)		
Employment, n (%)				1.000^d^	0.149
	Unemployed	23 (100)	22 (95.7)		
	Employed	0 (0)	1 (4.3)		

a Student *t* test.

b Chi-square test.

c Not applicable.

d Fisher exact test.

### Recruitment and Participation

**Figure 2.**
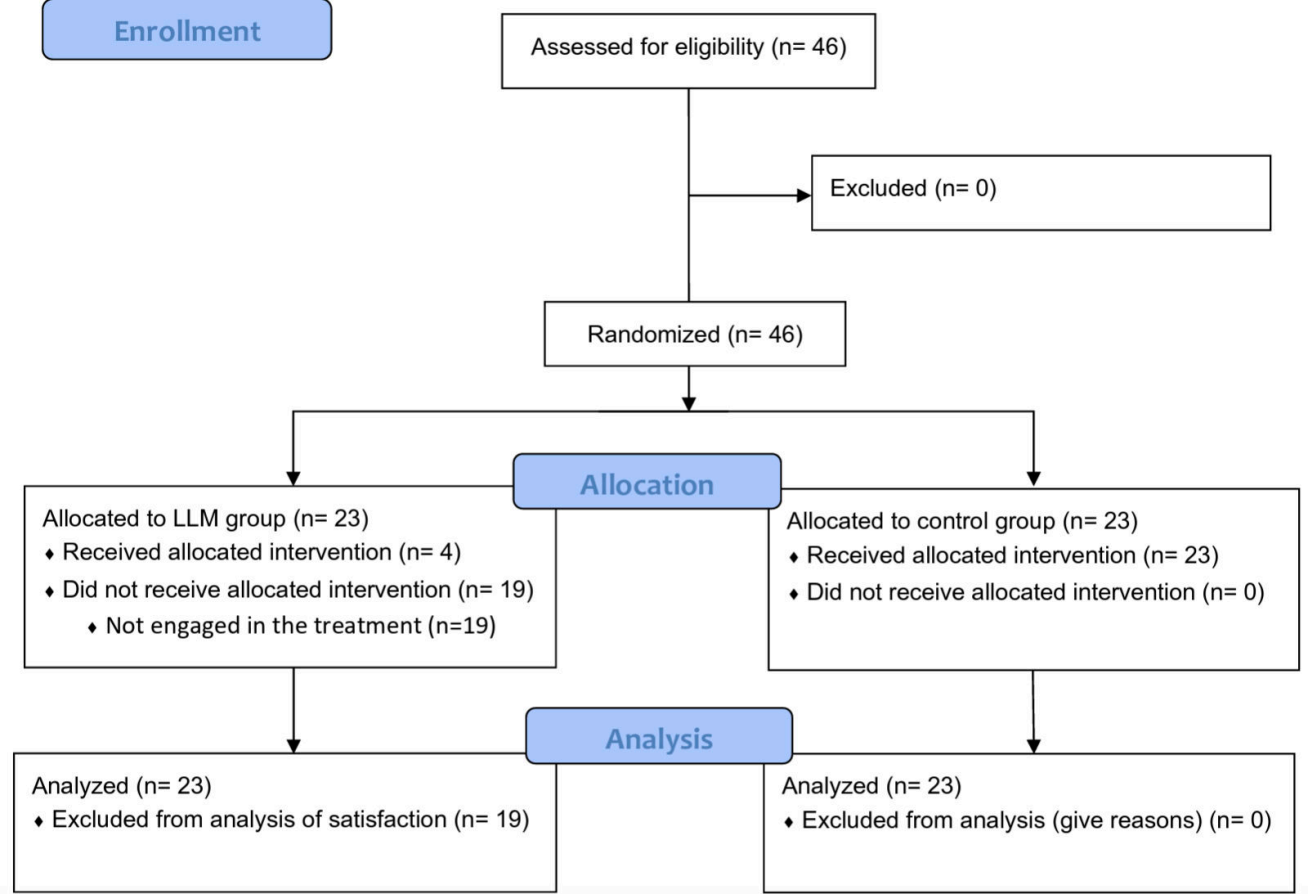
CONSORT (Consolidated Standards of Reporting Trials) flow diagram of the study.

All individuals agreed to participate in the study. All participants completed the initial digital competence questionnaire and the visual analog satisfaction scale at the end of the study. However, of the 23 students who were assigned to the ChatGPT group, only 4 (5.75%) of the students actively participated and interacted with the model for the resolution of the clinical cases. These 4 students completed the 4-week intervention and the final clinical case, but no additional students engaged with the LLM (Figure 2).

### Cost Analysis

The cost analysis of the intervention was calculated based on direct and indirect expenses. Direct costs included the OpenAI ChatGPT Team Plan (version 4.0) at US $26 per user/month. For 23 students over 4 weeks, this totaled US $530. Indirect costs accounted for (1) clinical case preparation: faculty spent 2 hours per 4 cases total designing LLM-compatible scenarios, equivalent to 8 hours at US $30.22/hour (average academic hourly rate), totaling US $60; (2) teacher training: a 4-hour workshop to familiarize educators with LLM tools, costing US $138; and (3) assessment time: the experimental group required additional time to evaluate LLM interaction logs (1 h/student), adding 23 hours (US $794). Total estimated cost: US $1738 (direct+indirect).

### Secondary Variables: Suitability of Outcome Measures

In terms of overall satisfaction, no significant differences were observed between the groups (*P*=.53), with a median of 77.00 (IQR 61.00‐84.00) for the control group and 77.00 (IQR 66.5‐85.5) for the intervention group. For this outcome, only four students in the experimental group who completed the trial were included for the analysis.

However, with respect to digital competencies, the item “Data analysis and management” showed a statistically significant difference between the groups, with a median of 6.00 (IQR 5.00‐6.75) in the control group and 7.00 (IQR 6.33‐7.30) in the intervention group (*P*=.042), with the intervention group standing out with higher skills in this area. Table 2 shows the overall data of these variables.

**Table 2.** Characteristics of outcome measures.

Variable	Control, median (IQR)	Intervention, median (IQR)	*P* value	Rosenthal R
Satisfaction	77 (61.00-84.00)	77.0 (66.5-85.5)	.71	0.056
Digital health literacy	7.30 (6.50-7.80)	7.30 (6.80-8.00)	.27	0.164
Effective management of scientific and health information	6.67 (5.67-7.00)	7.00 (6.33-7.67)	.15	0.211
Health communication 2.0	6.80 (5.80-7.40)	7.40 (6.60-7.80)	.27	0.162
Creation of scientific-health digital content	6.20 (5.20-7.00)	7.20 (6.40-7.60)	.05	0.286
Collective networking with health teams	6.50 (5.30-7.00)	7.00 (6.33-8.00)	.18	0.198
Data analysis and management	6.00 (5.00-6.75)	7.00 (6.33-7.30)	.04	0.299

## Discussion

### Principal Findings

The main objective of this study was to assess the feasibility of implementing ChatGPT as an LLM for improving clinical reasoning in physical therapy students. This is the first study that tries to implement the use of LLM for university students, and its findings offer preliminary insights into both the potential benefits and challenges associated with this innovative educational approach. It will serve as a foundation for designing a larger RCT that will assess the effectiveness of LLMs in enhancing clinical reasoning skills in physical therapy students.

The results obtained reflect some barriers for the implementation of this intervention in university centers, which must be addressed in future RCTs. While other studies have shown the effectiveness of ChatGPT for other health careers like ophthalmology [19], our study demonstrated a low active participation rate within the experimental group, with only 5.75% of students actively engaged with the model to solve clinical cases. The frequency of use of this tool for educational purposes in other studies with similar samples has been shown to be higher among students in other health care fields [20]. This suggests that while there is interest, the practical integration of LLMs into the learning routine of physical therapy students may face challenges, such as their comfort with new technologies, their perceived usefulness of the tool, or possible technical issues. One of the barriers identified by other studies is the absence of human interaction that can negatively affect students who prefer a personal connection with their educator, as shown in the study conducted by D’Mello et al [21].

Another issue for implementing LLM in physical therapy students could be the level of digital competencies shown by the participants, as, despite demonstrating a good level in the different items, in none of them was the average score excellent, and probably it may not be at a sufficient level to benefit from the use of this technology in a comfortable way for the learner. In fact, one of the main barriers to the implementation of this technology seems to be this lack of knowledge [16] in the field.

Specifically, the item “data analysis and management” was the one reported with the lowest score by the participants. It can therefore be identified that physical therapy students self-assess themselves as having a poor ability to use health databases and to handle analysis of large amounts of data [17]. While the rest of the health professions are making progress in this recent field of research, it is probably desirable that physical therapy universities change their teaching models so that their students are adapted to these new technologies once they finish their degree, which seems to be an issue in other health care careers [22].

The level of satisfaction with the study was high in both groups, with no significant differences between them. This fact provides a strong background to carry out the full study, since our hypothesis is that the overall satisfaction of students using LLM to improve their clinical reasoning will be higher than those using traditional methods, as we have already observed in other studies in medical students [23,24].

This statement is supported by the theoretical basis that, despite the low level of regular interaction within the LLM group (5/23, 5.75%), there were no differences between the groups. Thus, it could be inferred that low participation does not equate to low satisfaction, given that in both cases it was very similar. Therefore, we can consider two possible scenarios.

On the one hand, it might be that the students who did use the LLM found it so useful that it compensated for the dissatisfaction of those who did not use it. Alternatively, although few used it, those who did had no negative expectations, and the rest remained neutral.

Hence, the premise of the hypothesis arises from the notion of a latent or unexpressed acceptability, wherein—despite the low participation—there was no active rejection of the tool. This suggests that there is no psychological resistance to the use of LLMs as a learning method. There is a clear theoretical potential for students to adopt these tools once the previously mentioned barriers (lack of training, difficulty in understanding and managing interaction-derived data, insufficient usage time, etc) are overcome.

In consequence, we cannot currently assert superiority, nor can we dismiss its feasibility, given that the comparable level of satisfaction indicates that, under optimal conditions, LLMs could be accepted and integrated as a supplement to traditional methods.

Therefore, the intervention protocol appears to be valid and usable; however, it is important to better train the students in ChatGPT in order to obtain greater adherence in the intervention group. With the aim of improving the methodology, the entire study should be conducted for 8 weeks instead of the 4 weeks initially proposed. In this way, the first 4 weeks of the study would be dedicated to increasing the students’ knowledge and skills in the use of ChatGPT, followed by the 4 weeks of intervention. The cost of the study would therefore double from the US $530 initially proposed to a total of €920 (US $1074.24) to carry out the study, increasing the total number of weeks of the study to 8.

One of the main points highlighted in this pilot feasibility study is the low adherence of students to the LLM intervention, which suggests that pedagogical factors can significantly influence the acceptance and use of these tools in physical therapy education. The integration of technologies in the teaching of clinical reasoning requires not only access to the tool, but also adequate pedagogical preparation that fosters active learning and confidence in the use of AI [16]. Previous studies have shown that self-directed, simulation-based learning improves the acquisition of health science skills, provided that students understand their applicability and feel comfortable with the methodology [25]. In this sense, the incorporation of an analysis of the learning styles of the participants before the intervention would allow the introduction of the LLM to be personalized, increasing the adherence and effectiveness of the program. Tools such as Kolb’s Learning Style Inventory [26] or the VARK Learning Modality Preference Scale [27] have been successfully used to adapt pedagogical strategies in similar educational settings. As we have previously mentioned, for the future randomized clinical trial, we propose extending the intervention to eight weeks, incorporating an initial period of specific training on the use of LLMs and their application in clinical reasoning, which could improve participation and the impact of the tool in the training of physical therapy students.

### Limitations

This study has several limitations. One of the main limitations of this study was the lack of a structured evaluation of the barriers perceived by the students who participated in the experimental group. While the initial objective was to evaluate the feasibility of the intervention, future research should include a formal evaluation of the obstacles encountered by students to improve adherence to the tool. Through informal conversations with participants, factors such as lack of familiarity with AI, preference for human interaction, perception of additional workload, and technological difficulties were identified. In a future RCT, questionnaires and semistructured interviews will be included to identify and mitigate these factors, ensuring that the intervention can be optimally implemented.

Also, the small sample size and short duration limit the generalizability of the findings. The low engagement rate in the experimental group also poses a significant challenge to interpreting the results. In addition, students were not blinded to group allocation, which could introduce bias. Despite these limitations, the study provides valuable preliminary data on the feasibility and potential impact of LLMs in physical therapy education.

### Conclusions

This pilot study investigated the feasibility of using LLMs like GPT-4 to enhance clinical reasoning skills in physical therapy students. While recruitment and participation were high, actual engagement with the LLM was low, so strategies for increasing the engagement should be implemented to carry on the complete study.

The cost of providing ChatGPT Premium for four weeks was US $530, raising questions about cost-effectiveness given the low engagement. However, future studies should consider a training period and indirect costs, increasing the total amount to US $1738.

## Supplementary material

10.2196/66126 Multimedia Appendix 1 Clinical cases example.

10.2196/66126 Multimedia Appendix 2 Digital competencies questionnaire in English.

10.2196/66126 Checklist 1 CONSORT (Consolidated Standards of Reporting Trials) checklist.

## Abbreviations

AI: artificial intelligence
CONSORT: Consolidated Standards of Reporting Trials
ICF: International Classification of Functioning, Disability and Health
LCHUS: La Salle Centre for Higher University Studies
LLM: large language model
RCT: randomized controlled trial
WHO: World Health Organization
