# Supplementary material for: Feasibility of a Randomized Controlled Trial of Large AI-Based Linguistic Models for Clinical Reasoning Training of Physical Therapy Students: Pilot Randomized Parallel-Group Study
Source: JMIR Form Res. 2025 Jul 23;9:e66126. doi: 10.2196/66126 (PMC12329153; doi:10.2196/66126)
Supplement: Multimedia Appendix 2 [file formative-v9-e66126-s002.docx]

**Appendix 2. Digital Competences questionnaire (English version)**

**Digital competence number 1: Digital Health Literacy**

**«Knowledge of the digital ecosystem and gaining the basic skills to appropriately and safely use different digital devices and applications for health purposes».**

- 1. Do you know the different devices, software, formats, types of documents, digital tools and web platforms needed to develop your daily work activity?

1.2. Do you consider that you have the operational skills and basic computer skills to perform the computer activities necessary in your daily life, such as web browsing, e-mail management, use of word processors, spreadsheets, databases, information storage, etc.?

1.3. Are you able to act ethically and safely on the Internet, protecting your privacy and managing your passwords appropriately, and are you aware of the potential risks involved in communicating via electronic media in the workplace?

1.4. Do you feel confident in your ability to identify, analyse and critically evaluate health websites, autonomously differentiating those that are reliable and of high quality from those that are not?

**Digital competence number 2: Effective management of scientific and health information**

**«Ability to manage scientific information and knowledge in an effective way to make decisions based on scientific evidence and to improve patient care».**

2.1. Do you know the different channels through which digital information circulates and learn how to search, filter, select, monitor, evaluate, capture, store, organise and retrieve it efficiently?

2.2. Do you think you have the ability to process information systematically and maintain a critical attitude that allows you to add value to information through content curation and share it with the community?

2.3. Do you feel confident in your ability to recognise the criteria of quality, validity and reliability of the information available on the Internet, and also to be aware of the legal and ethical principles that should govern the use of ICT?

**Digital competence number 3: Health communication 2.0**

**«Use of digital technologies, devices and channels in an appropriate way to improve networking and non-face-to-face communication between health workers**».

3.1. Do you feel able to adequately manage your digital identity and reputation in the health context?

3.2. Are you familiar with the different communities and networks for professional purposes that can be used in the field of health?

3.3. Do you consider that you have the ability to appropriately use the new channels and languages of digital communication in health, as well as to manage your digital identity and behave appropriately in the different digital contexts related to cyberethics, cybersecurity, privacy and data protection regulations?

3.4. Are you able to connect, interact and converse across different digital platforms and tools used in healthcare settings?

3.5. Do you know how to use the web to publish and disseminate digital content of value in health, with the aim of capturing the attention of the people you are targeting?

**Digital competence number 4: Creation of scientific-health digital content.**

**«Harnessing ICT to facilitate and enhance scientific research and publication 2.0, as well as the design and production of digital health content**».

4.1. Do you consider yourself able to understand the different types of digital content that exist and the tools that enable them to be produced?

4.2. Are you familiar with intellectual property, copyright and application licences for digital content?

4.3. Do you think you know how to correctly use the different digital content creation tools to design and produce scientific-health content in digital environments, facilitating the transfer of knowledge and providing added value, including for scientific research and publication?

4.4. Have you acquired basic knowledge of the design and production of digital content that encourages the participation of the target audience, such as patients, citizens or other professionals?

4.5. Do you feel able to satisfy your individual creativity and solve day-to-day problems by creating digital content, targeting it appropriately to the target audience and disseminating it online?

**Digital competence 5: Collaborative networking with health teams**

**«Use of digital tools and resources that facilitate the development of interdisciplinary projects and non-face-to-face healthcare in collaboration with different healthcare agents, including patients**».

5.1. Do you feel able to know and use the different digital tools based on ‘cloud’ technology that facilitate non-physical cooperation and teamwork?

5.2. Are you able to detect and take advantage of the different collaborative health networks generated on the Internet, and do you know how to contribute appropriately to them?

5.3. Do you think you have the ability to connect, converse, share knowledge and cooperate with other professionals in a non-face-to-face way through the Internet, with a common goal?

**Digital competence number 6: Data analysis and management**

**«Knowledge of the different sources of health data and how to process them to extract knowledge and results in real time to facilitate clinical decision-making**».

6.1. Do you consider yourself capable of knowing the most relevant sources of health data and information in the health environment?

6.2. Do you have the necessary knowledge and skills to carry out advanced database management using the usual office automation tools?

6.3. Do you think you know how to collect, organise, analyse, interpret and use data and clinical information obtained from different data sources?

6.4. Do you have the ability to select or design optimal indicators for the analysis of health outcomes and the different internal processes of your area of knowledge or clinical service?

6.5. Do you feel able to extract knowledge from the large volumes of big data generated in each clinical setting, applying the principles of evidence-based medicine?

6.6. Are you familiar with the regulatory framework and do you understand the importance of security, privacy and confidentiality of health data in the digital environment?

TOTALLY UNABLE TOTALLY ABLE

0 1 2 3 4 5 6 7 8 9 10

UNFAMILIARY FAMILIAR

0 1 2 3 4 5 6 7 8 9 10
